# Oncolytic adenovirus H101 enhances the anti-tumor effects of PD-1 blockade via CD47 downregulation in tumor cells

**DOI:** 10.32604/or.2024.055746

**Published:** 2025-04-18

**Authors:** CHENXIAO QIAO, YIPENG XU, YEDIE HE, ZHIJIAN CAI, HUA WANG

**Affiliations:** 1 Institute of Immunology, Zhejiang University School of Medicine, Hangzhou, 310058, China; 2 Department of Urology, Zhejiang Cancer Hospital, Hangzhou, 310022, China

**Keywords:** Cancer, Oncolytic adenovirus, Macrophage, CD47, Programmed cell death protein 1 blockade (PD-1 blockade)

## Abstract

**Objective:**

To investigate the anti-tumor effects of an E1B55KD-deleted oncolytic adenovirus, H101, in combination with a humanized anti-PD-1 (Programmed cell death protein 1) monoclonal antibody, Camrelizumab.

**Methods:**

Anti-tumor efficacy of intratumoral injection of H101 or/and intraperitoneal injection of Camrelizumab were evaluated in an immune system humanized NOD *Prkdc*^*scid*^ *Il2rg*^*-/-*^ mice subcutaneous (S.C.) tumor model, established with human glioblastoma of unknown origin cell line U87-MG, and human bladder cancer cell line T24 and YTS-1. The mechanism by which H101 induced anti-tumor immunity were also investigated.

**Results:**

Combining H101 with Camrelizumab demonstrated more potent anti-tumor effects than monotherapy in mouse S.C. tumor model. Increased tumor-infiltrating T cells were observed in the combined treatment group. H101 infection decreased the expression of CD47 in cancer cells, thereby promoting macrophages to phagocytose cancer cells. Following the H101-mediated activation of macrophages, increased levels of cytokines, including TNF, IL-12 and IFN-γ were observed. Moreover, when induced THP-1 cells were co-cultured with H101-treated cancer cells, expression of IFN-γ was increased in T cells. Elimination of IL-12 using an anti-IL-12 antibody abolished IFN-γ production from T cells. In addition, infection with H101 increased PD-L1 expression in YTS-1 cells. These results suggested that H101 may act synergistically to enhance the therapeutic efficacy of PD-1 blockade in cancer via suppressing CD47 signaling, which may promote macrophages to phagocytose tumor cells and activate CD8^+^ T cells.

**Conclusion:**

The combination of H101 with PD-1 blockade exhibits potential as a novel strategy for the treatment of cancer.

## Introduction

In 2022, ~20 million new cancer cases and 9.7 million cancer-related deaths occurred [1]. The majority of patients with locally advanced or metastatic cancer are incurable with currently available treatment options. Immune checkpoint inhibitors alone or combined with conventional treatment options, such as radiotherapy and chemotherapy, have become an increasingly used therapeutic option for numerous patients with advanced or metastatic cancer [2]. However, the majority of patients remain resistant to these treatments [3]. Biomarkers that predict the response and development of combined treatment options may improve the efficacy of immunotherapy, and these are required to improve treatment efficacy in larger patient populations. Results of previous studies demonstrated that tumor response was associated with the expression of PD-L1 [4] and the increase of tumor-infiltrating CD8^+^ T cells [5]. Thus, inducing PD-L1 upregulation and/or attracting CD8^+^ T cells into tumors through altering the immune-suppressive tumor microenvironment may exhibit potential in enhancing the efficacy of immune checkpoint blockade.

Oncolytic adenoviruses with deletion of E1B-55kD or mutated E1A replicate selectively to destroy cancer cells that have deficient p53 or a disrupted Rb pathway. Notably, healthy cells are unaffected by this process, which is known as oncolytic virotherapy. Thus, this process exhibits potential as a novel treatment for cancer [6]. However, a phase I/II trial of ONYX-015, an E1B55 KD-deleted adenovirus, included delivery via injection into the primary tumor to treat pancreatic carcinomas. Results of this study demonstrated modest anti-tumor activity, particularly following the use of ONYX-015 as a single agent; however, this treatment is feasible and generally well tolerated either alone or in combination with gemcitabine [7]. Moreover, phase I trials of an E1A-mutated oncolytic adenovirus, Delta-24-RGD, have been completed in patients with recurrent high-grade gliomas. Results of this study demonstrated that the virus was well tolerated [8]. Over the past two decades, the results of numerous preclinical studies demonstrated potent anti-tumor effects of oncolytic adenoviruses in cancer. However, clinical applications in patients with cancer remain limited, due to limited therapeutic efficacy when used as a monotherapy [9]. Results of a previous study demonstrated that oncolytic viruses induced infected cells to lyse, which may stimulate systemic anti-tumor immunity through the release of danger-associated molecular patterns, pathogen-associated molecular pattern molecules and cytokines. This process leads to the maturation of antigen-presenting cells, which recruit CD4^+^ and CD8^+^ cells to destroy cells expressing viral antigens on tumors [10]. In addition, results of a previous study demonstrated that Newcastle disease virus (NDV) induced PD-L1 upregulation in both infected tumors and tumors not infected by the virus, and intratumoral therapy with NDV sensitized the tumors to the efficacy of programmed cell death protein 1 (PD-1) and programmed cell death ligand 1 (PD-L1) blockade in tumor models [11].

Thus, the present study aimed to investigate the anti-tumor effects of H101, an E1B55KD and partial E3 deleted oncolytic adenovirus. Notably, this is the first and only adenovirus that was approved by the China State Food and Drug Administration in 2005 for the treatment of head and neck cancer, in combination with a humanized anti-PD-1 monoclonal antibody, Camrelizumab (SHR-1210). Our results demonstrated that H101 infection induced downregulation of CD47 in infected cancer cells, which promoted macrophages to phagocytose cancer cells. *In vivo*, the combined therapy of H101 with Camrelizumab demonstrated more potent anti-tumor effects than a single agent alone. Following analysis of treated tumors, we observed an increased number of tumor-infiltrating T cells, particularly IFN-γ-expressing CD8^+^ T cells, in the combined treatment group. Moreover, increased expression levels of cytokines, including TNF, IL-12 and IFN-γ, were observed in H101-treated or combination-treated tumor tissues. These results suggested that H101 may indirectly activate macrophages, and subsequently induce the activation of T cells.

## Materials and Methods

### Cell lines

Human glioblastoma of unknown origin cell line U87-MG, human myeloid leukemia mononuclear cells THP-1, and human bladder cancer cell line T24 were purchased from the American Type Culture Collection (ATCC). Human bladder cancer cell line YTS-1 [12,13] was provided by the Department of Urology, Tohoku University School of Medicine (Sendai, Japan). Human embryonic kidney 293 (HEK-293) cells were obtained from the Cell Bank of Chinese Academy of Sciences (Shanghai, China).

T24 and YTS-1 cells were cultured in RPMI-1640 medium (Shanghai BasalMedia Technologies Co., Ltd., L210KJ, Shanghai, China) with 10% fetal bovine serum (FBS, Yeasen, 40130ES76, Shanghai, China) and 1% antibiotic solution (100 U/mL penicillin, 100 μg/mL streptomycin sulfates, CellMax, CPS101.02, Beijing, China). U87-MG and HEK-293 cells were cultured in Dulbecco’s modified Eagle’s medium (DMEM, Shanghai BasalMedia Technologies Co., Ltd., L113KJ, Shanghai, China) supplemented with 10% FBS (Yeasen, 40130ES76) and 1% antibiotic solution (CellMax, CPS101.02). THP-1 cells were cultured in RPMI-1640 medium (Shanghai BasalMedia Technologies Co., Ltd., L210KJ) supplemented with 10% FBS (Yeasen, 40130ES76), 1% antibiotic solution (CellMax, CPS101.02) and β-mercaptoethanol (0.05 mM, Shanghai BasalMedia Technologies Co., Ltd., S110JV, Shanghai, China). Induced THP-1 cells were stimulated with 50 ng/mL phorbol 12-myristate 13-acetate (Sigma-Aldrich, Merck KGaA, P8139, Darmstadt, Hessen, Germany) for 12 h to induce M0 macrophages, which were cultured in RPMI-1640 medium (Shanghai BasalMedia Technologies Co., Ltd., L210KJ) supplemented with 10% FBS (Yeasen, 40130ES76), 1% antibiotic solution (CellMax, CPS101.02) and β-mercaptoethanol. All cells were maintained in a humidified incubator at 37°C with 5% (v/v) CO_2_. All the cells used were tested for presence of mycoplasma, and the test results were negative.

### Recombinant adenovirus

H101, a recombinant type 5 human adenovirus (also known as Oncorine) with a E1B55KD deletion and partial E3 region which can selectively replicate in tumor cells [14] was purchased from Shanghai Sunway Biotech Co., Ltd., Shanghai, China. Viruses were transfected into HEK293 cells to produce adenoviruses. Brefly, HEK293 cells grew to 80% confluent and were infected with viruses at MOI of 30, and cultured with DMEM supplemented with 5% FBS and 1% antibiotic solution for 5 days. Cells were harvested and frozen-thawed 6 times, centrifuged at 3000 rpm (Thermo Scientific Sorvall ST 40R Centrifuge) for 10 min, and the supernatant was collected for use. Viral titer was determined using a standard plaque assay.

### PD-1 inhibitor

Camrelizumab (approval number: S20190027) is the earliest humanized immunoglobulin G4 (Ig G4) monoclonal anti-PD-1 antibody to be marketed in China for the treatment of various tumors [15], which was purchased from Jiangsu Hengrui Medicine Co., Ltd., Suzhou, China.

### Western blot analysis

Cells were collected and washed twice with ice-cold 1 × PBS. Cells were lysed using RIPA lysis buffer (Beyotime Institute of Biotechnology, P0013B, Shanghai, China) and 1 mM PMSF (Solarbio, P0100, Beijing, China) was added on ice for 30 min. Cell lysates were separated via SDS-PAGE on a 12% gel, and proteins were subsequently transferred onto PVDF membranes (MilliporeSigma, ISEQ00010, Darmstadt, Hessen, Germany). Membranes were blocked with 5% BSA in PBS-Tween-20 (PBS-T, Solarbio, P1010) buffer and incubated with anti-coxsackie-adenovirus receptor (CAR, Proteintech Group, Inc., Chicago, IL, USA, 11777-1-AP) at a concentration of 1.5 μg/mL or anti-GAPDH (Shanghai Abways Biotechnology Co., Ltd., AB0036, Shanghai, China) at a dilution of 1:5000 at 4°C overnight. Following primary incubation, membranes were washed with PBS-T buffer three times, and subsequently incubated with goat anti-rabbit IgG (H+L) HRP antibody (MultiSciences Biotech Co., Ltd., GAR007, Hangzhou, China) diluted at 1:5000 for 1 h at room temperature. Protein bands were visualized using UltraSignal ECL Western Blotting Detection Reagent (Beijing 4A Biotech Co., Ltd., 4AW011-1000, Beijing, China). Membranes were subsequently scanned using the Tanon 4500 Gel Imaging System (Tanon, Shanghai, China).

### Cell apoptosis assay

U87-MG, YTS-1 or T24 cells were collected and washed with 1 × PBS. Cell apoptosis was determined using an Annexin V-APC/PI apoptosis kit (cat. no. AP107; MultiSciences Biotech Co., Ltd.), according to the manufacturer’s instructions. Briefly, Cells were collected in 1.5 mL tubes and washed 3 times with 1 × PBS. Then cells were resuspended in 100 μL 1 × binding buffer, stained with 5 μL Annexin V-APC and 5 μL PI Staining Solution for 10 min at room temperature, protected from light. Add 400 μL 1 × Binding Buffer, then cells were detected on CytoFlex flow cytometer (Beckman Coulter, Inc., Brea, CA, USA). Early apoptosis cells were Annexin V+ PI−. Late apoptosis cells were Annexin V+ PI+.

### Animal studies

Female NOD *Prkdc*^*scid*^ *Il2rg*^*-/-*^ (NSG) mice (age, 6 weeks) were purchased from GemPharmatech Co., Ltd., Nanjing, China. Mice were housed under specific pathogen-free conditions, and the Animal Care and Use Committee of Zhejiang University approved all experimental protocols (approval no. ZJU20230232). Mice were housed in a controlled environment (12 h light/12 h dark cycle) with *ad libitum* access to standard food and water, and were monitored every day. Mice were subcutaneously injected with 3 × 10^6^ U87-MG cells, and 5 × 10^6^ T24 or YTS-1 cells on Day 0. Cells cultured in 10 cm dish were collected and resuspended with 2 mL 1 × PBS. Diluted cells at 1:100 with 1 × PBS. Transfer 10 μL of cell suspension on the blood counting chamber. Cells were counted with microscope. Cells number = counted cells number/4 × 10^4^ × 100 × 2. Then tumor cells were resuspended in 1 × PBS, diluted to 5 × 10^7^ cells/mL. Tumor-bearing mice received H101 treatment (2.5 × 10^7^ pfu per tumor) via intratumoral injection every two days, and tumor size was measured every day with vernier caliper, and tumor sizes were recorded when the tumor volume reached 100 mm^3^. Due to the significant tumor shrinkage caused by camrelizumab, tumors in early stage treated with camrelizumab were too small to be accurately injected with the virus into the tumor. Thus, for the combination therapy, tumor-bearing mice received oncolytic viral treatment (2.5 × 10^7^ pfu per tumor) via intratumoral injection every two days and/or received 50 μg Camrelizumab treatment via intraperitoneal injection every four days according to the internal pharmacodynamic study documents from pharmaceutical manufacturers, when the tumor volume reached 300 mm^3^. In specific experiments, NSG mice were also injected with U87-MG, T24 or YTS-1 cells and 1 × 10^6^ human peripheral blood mononuclear cells (PBMCs) isolated from the peripheral blood of healthy volunteers, which were approved with Zhejiang Cancer Hospital (approval no. zjzlsd2021-12-004). According to the manufacturer’s instructions, PBMCs were isolated with a Lymphocyte Separation Medium (cat. no. LTS1077; TBDsciences, Tianjin, China). Tumor sizes were measured using Vernier calipers every two days, and tumor volume was calculated using the following formula: Volume = largest dimension × smallest dimension^2^ × 0.5. A total of 90 mice with subcutaneous tumor were randomly divided into 18 groups, and each group included five mice. The mice were then administered an intratumoral injection of H101 and/or intraperitoneal injection camrelizumab. Animals were sacrificed using cervical dislocation under anesthesia with intraperitoneal injected of 50 mg/kg pentobarbital sodium when the tumor diameter reached 20 mm. Death was confirmed when the heart rate of the animal had completely stopped, and the pupils were dilated.

### Immunofluorescence and confocal microscopy

Following inoculation, tumor tissues were isolated on Day 14. Tissues were embedded in Tissue-Tek® OCT Compound, and 5-μm-thick frozen sections were cut using CryoStar NX50 (Thermo Fisher Scientific, Inc., Waltham, MA, USA). Subsequently, all sections were fixed using −20°C prechilled methyl alcohol for 10 min and permeabilized using 0.1% Triton-X-100. Following blocking with 5% BSA (Sangon, 9048-46-8, Shanghai, China) and 3% goat serum (Yeasen, 36119ES10) in 1 × PBS, proteins were incubated with mouse anti-CD8 (cat. no. 66868-1-Ig; Proteintech Group, Inc., Wuhan, China) and rabbit anti-IFN-γ (cat. no. 15365-1-AP; Proteintech Group, Inc.) overnight at 4°C. Subsequently, cells were washed three times with 1 × PBS and incubated with IFLUOR™ 488-(HUABIO, HA1211, 1:500) and IFLUOR™ 549-(HUABIO, HA1126, 1:500)-labeled secondary antibodies for 30 min at room temperature. Following washing three times with 1 × PBS, nuclei were stained with DAPI (cat. no. D3571; Invitrogen; Thermo Fisher Scientific, Inc.). Fluorescent signals were detected using an Olympus IX83 FV3000 confocal microscope (Olympus, Tokyo, Japan). Quantitative fluorescence signals were analysed by ImageJ (National Institutes of Health, Java 1.8.0-345, Bethesda, MD, USA).

### Quantitative PCR (qPCR)

Total RNA was extracted from cells using RNAiso plus (cat. no. 9109; Takara Bio, Inc., Kyoto, Japan). In total, 600 ng RNA was reverse transcribed into cDNA using the HiScript® II Q RT SuperMix (cat. no. R223-01; Vazyme Biotech Co., Ltd., Nanjing, China) following the manufacturer’s instructions. Keep the mixture under 42°C for 30 min. qPCR was conducted using ChamQ Universal SYBR qPCR Master Mix (cat. no. Q711-02; Vazyme Biotech Co., Ltd.) and the CFX96 Touch Real-Time PCR Detection System (Bio-Rad Laboratories, Inc., Hercules, CA, USA). PCR primer sequences are shown in Table 1. The following thermocycling conditions were used for qPCR: Initial denaturation at 95°C for 30 s, followed by 40 cycles at 95°C for 5 s and 60°C for 34 s. mRNA levels were analyzed using the 2^−ΔΔCt^ method. Use hACTB as reference genes.

**Table 1:** Sequences of primers for reverse transcription-quantitative PCR

Gene	Primer sequence
hCD47 F	5′-TATCCTCGCTGTGGTTGGACTG-3′
hCD47 R	5′-TAGTCCAAGTAATTGTGCTAGAGC-3′
hICAM1 F	5′-AGCGGCTGACGTGTGCAGTAAT-3′
hICAM1 R	5′-TCTGAGACCTCTGGCTTCGTCA-3′
hFAS F	5′-GGACCCAGAATACCAAGTGCAG-3′
hFAS R	5′-GTTGCTGGTGAGTGTGCATTCC-3′
Hpecam F	5′-AAGTGGAGTCCAGCCGCATATC-3′
Hpecam R	5′-ATGGAGCAGGACAGGTTCAGTC-3′
hHLA-A F	5′-AGATACACCTGCCATGTGCAGC-3′
hHLA-A R	5′-GATCACAGCTCCAAGGAGAACC-3′
Hactb F	5′-CACCATTGGCAATGAGCGGTTC-3′
Hactb R	5′-AGGTCTTTGCGGATGTCCACGT-3′

### Phagocytosis assay

Tumor cells were stimulated with oncolytic viruses at a multiplicity of infection of 1 for 24 h prior to collection. Cells were stained with 5,6-carboxyfluorescein diacetate succinimidyl ester (CFSE, Invitrogen, C34570, Thermo Fisher Scientific, Inc.) according to the manufacturer’s instructions. CFSE-labeled cells at 3 × 10^5^ cells per well were seeded into 6-well plates and co-cultured with induced THP-1 cells (1 × 10^5^ cells per well) at a ratio of 3:1 under 37°C. After 8 h, all cells were collected and stained with anti-human CD45 (BioLegend, Inc., 982304, San Diego, CA, USA) to distinguish induced THP-1 from tumor cells. Subsequently, CFSE-labeled CD45^+^ induced THP-1 cells were detected using a CytoFlex flow cytometer (Beckman Coulter, Inc.). Anti-CD47 (Bio X cell, BE0019-1;) was added to the medium at a concentration of 10 μg/mL.

### Measurement of cytokine levels

The concentrations of cytokine levels of induced THP-1 and tumor tissue were analyzed using ELISA kits according to the manufacturer’s protocols. ELISA kits for the detection of human IFN-γ (cat. no. 430104), IL-12 (cat. no. 431704) and TNF (cat. no. 430204) were purchased from BioLegend, Inc. Briefly, sample supernatants were added to pre-coated ELISA plates along with a dilution reagent and incubated for 2 h at room temperature. After incubation, the wells were washed, and diluted detection antibodies were added. Following additional aspiration and washing steps, streptavidin-HRP solution was applied to each well, and the plate was incubated in the dark for the designated time. After further washes, substrate solution was added and incubated for 20 min at room temperature. The reaction was stopped by adding the stop solution, and the optical density (OD) was read at 450 nm using a microplate reader (Thermo Fisher Scientific, Thermo Scientific 5250040). Cytokine concentrations for IFN-γ, IL-12, and TNF were then calculated based on the standard curves.

### Flow cytometry

Cells were collected and washed three times with 1 × PBS. Following the staining of surface markers using corresponding antibodies at room temperature for 20 min, cells were permeabilized using IC Fixation Buffer (Thermo Fisher Scientific, Inc., 00-8222) and stained for the detection of intracellular cytokines at room temperature for 30 min. Flow cytometry was performed on a CytoFlex flow cytometer, and data were analyzed using FlowJo software v10.6.2 (TreeStar Ashland). The following antibodies (1 µL antibody was used for staining 1 × 10^7^ cells) were used to stain cells: Fixable Viable Dye eFluorTM 520 (cat. no. 65-0867-14; Invitrogen; Thermo Fisher Scientific, Inc.), phycoerythrin-conjugated anti-human CD8a (BioLegend, Inc., 301007), allophycocyanin-conjugated anti-human IFN-γ (BioLegend, Inc., 502511) and FITC anti-human CD45 Antibody(BioLegend, Inc., 368507).

### Immunohistochemical (IHC) analysis

Tumor tissues were isolated on Day 7 following treatment. Paraformaldehyde-fixed paraffin-embedded murine tumor tissue samples were cut into 4-µm-thick sections and heated in an oven at 60°C for 90 min. The slides were then placed in xylene (5 min/time, 3 times) for deparaffinization, followed by hydration in a graded series of ethanol solutions (100%, 95%, and 75%) and washed with phosphate-buffered saline (PBS, 3 times). For antigen retrieval, the slides were immersed in 10 mM sodium citrate buffer (pH 6.0) and heated at 95°C for 10 min, then allowed to cool naturally. After washing with PBS, the slides were incubated with 3% hydrogen peroxide (H_2_O_2_) for 10 min at room temperature to block endogenous peroxidase activity. Subsequently, the slides were treated with Triton-PBS for 5 min to permeabilize the tissue, and then blocked with 5% BSA for 30 min at room temperature. Then slides were stained with anti-IFN-γ (Proteintech, Inc., 15365-1-AP;) primary antibody at 4°C overnight. The following day, the sections were washed 3 times with PBS (10 min each), and treated with HRP-conjugated secondary antibodies for 30 min at room temperature. After washing with PBS, 3,3′-diaminobenzidine (DAB) reagent was added dropwise for color development, and the reaction was stopped by rinsing with tap water. The slides were counterstained with hematoxylin for 1 min, followed by differentiation in 1% hydrochloric acid alcohol solution. Finally, the sections were dehydrated, cleared, and mounted with neutral balsam. Images of the sections were acquired using a BX63 Olympus microscope (Olympus Corporation, Kyoto, Japan).

### Statistical analysis

All statistical analyses were performed using GraphPad Prism 8.0 (GraphPad Software, Inc., San Diego, CA, USA). All data are expressed as the mean ± standard deviation. Unpaired Student’s *t*-tests were used to compare differences between two groups. One-way ANOVA followed by Tukey’s *post hoc* test was used to compare differences among multiple groups. The log-rank test was used for survival analysis. *p* < 0.05 was considered to indicate a statistically significant difference.

## Results

### H101 inhibits tumor growth in a CAR-dependent manner

**Figure 1:**
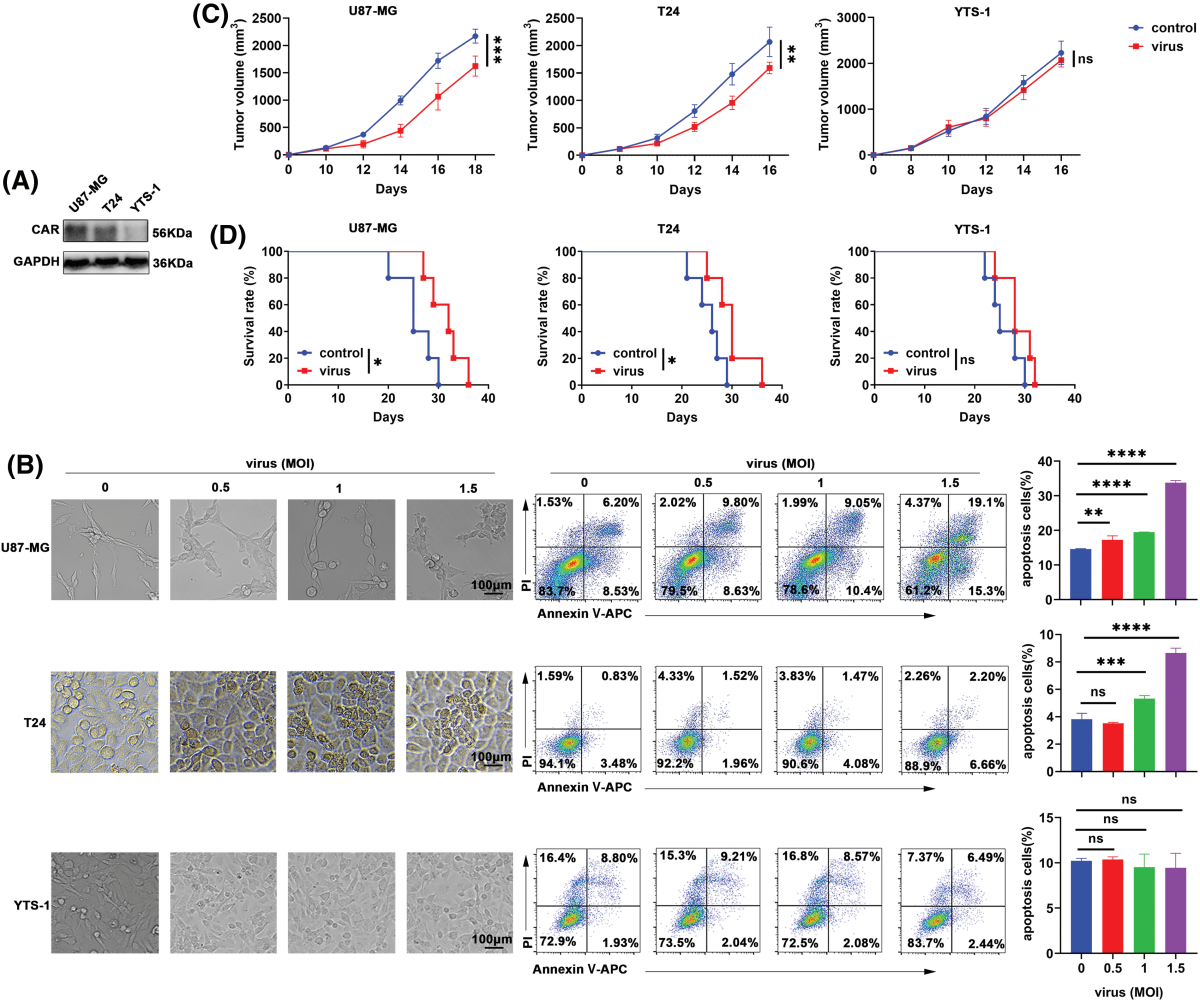
H101 suppresses tumor growth through promoting cancer cell apoptosis. (A) Western blot analysis of CAR (55 kDa) expression in U87-MG, T24 and YTS-1 cells. (B) U87-MG, T24 and YTS-1 cells were stimulated with oncolytic viruses at a multiplicity of infection of 0.5, 1 or 1.5 for 24 h. Representative images demonstrated the morphology of these cells. Apoptotic cells were stained with annexin V/PI and analyzed using flow cytometry (n = 3). (C) Tumor sizes and (D) survival of tumor-bearing NSG mice inoculated with U87-MG, T24 and YTS-1 cells that received an intratumoral injection of physiological saline solution or H101 (2.5 × 10^7^ pfu per tumor). Representative results from three independent experiments are shown (n = 5). ns, not significant, *p < 0.05, **p < 0.01, ***p < 0.001, ****p < 0.0001. MOI, multiplicity of infection; NSG, NOD *Prkdc*^*scid*^ *Il2rg*^*-/-*^; CAR, coxsackie-adenovirus receptor.

Adenoviral infection begins with the high-affinity binding of fibrin to the CAR on the cell surface. Following initial interaction with the CAR, an exposed RGD domain on the adenoviral subunit binds to the cell surface α_v_ integrin to trigger endocytosis, which internalizes viral particles. Subsequently, internalized adenoviruses replicate in tumor cells and this, in turn, induces oncolysis [16]. Therefore, CAR-positive and CAR-negative cancer cell lines were used in the present study to determine the anti-tumor effects of H101. As shown in Fig. 1A, U87-MG cells exhibited a high expression of CAR, T24 cells exhibited a moderate CAR expression, while YTS-1 cells exhibited low levels of CAR expression. In addition, H101 induced apoptosis of U87-MG and T24 cells in a dose-dependent manner, and apoptosis increased in U87-MG cells (Fig. 1B). However, H101 did not affect the apoptosis of CAR-negative cell line, YTS-1 (Fig. 1B). Consistent with these results, our study also demonstrated that H101 significantly inhibited the growth of U87-MG and T24 tumors, and prolonged the survival of the corresponding tumor-bearing mice (Fig. 1C,D). However, H101 did not exert anti-tumor effects in YTS-1 tumor-bearing mice (Fig. 1C,D). These results indicated that H101 inhibited tumor growth, and this was dependent on CAR expression.

### H101 synergizes with anti-PD1 to enhance anti-tumor immunity

**Figure 2:**
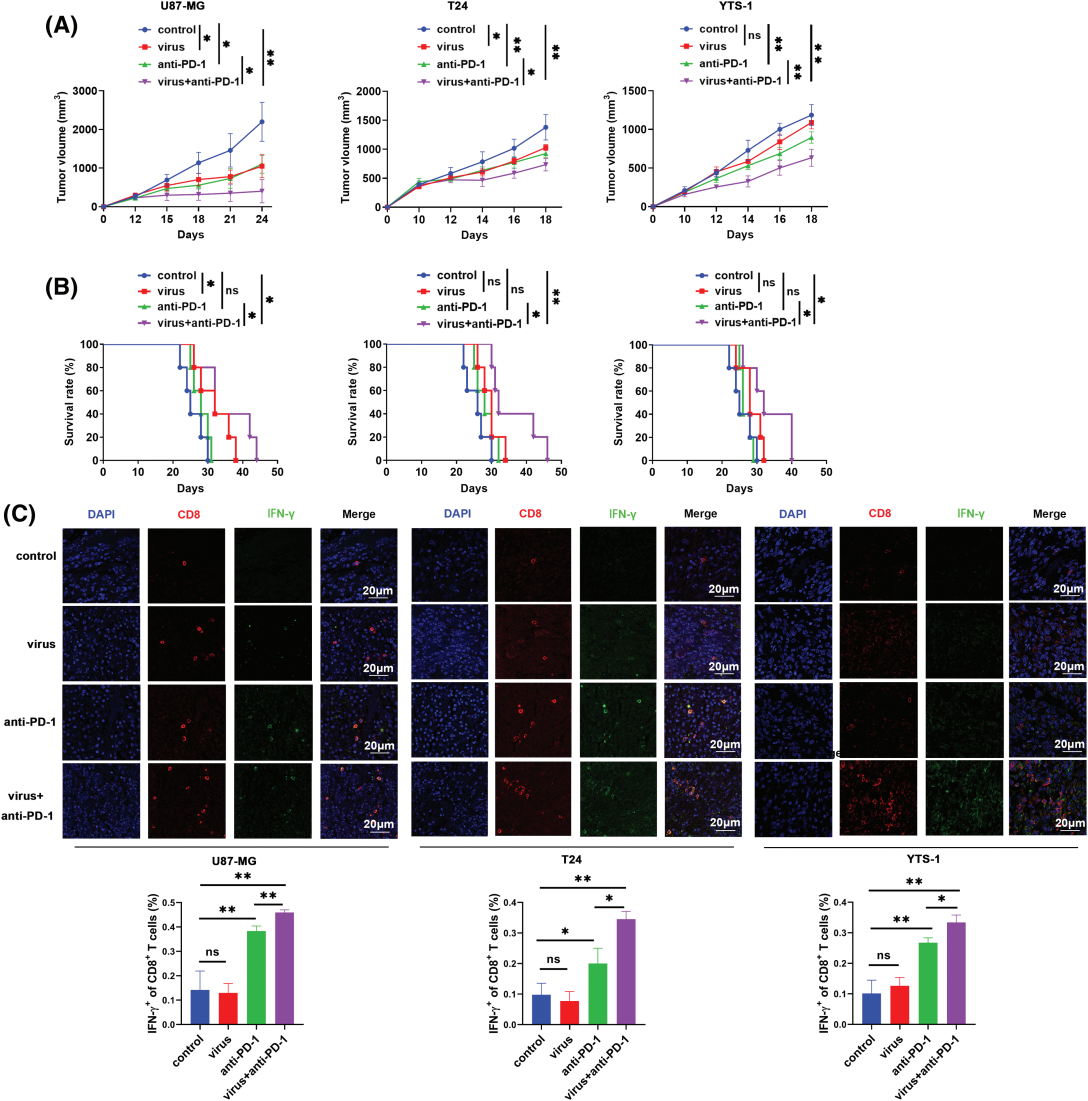
H101 enhances the anti-tumor effects of anti-PD-1 via increasing IFN-γ. (A) Tumor sizes and (B) survival of U87-MG, T24 and YTS-1 tumor-bearing NSG mice that received a transfer of 1 × 10^6^ PBMCs and intratumoral injection of H101 (2.5 × 10^7^ pfu per tumor) every two days, along with an intraperitoneal injection of 50 μg Camrelizumab every four days. (C) Immunofluorescence analysis of IFN-γ in tumor-infiltrating CD8^+^ T cells of U87-MG, T24 and YTS-1 tumor-bearing mice that received an intratumoral injection of H101 (2.5 × 10^7^ pfu per tumor) every two days along with intraperitoneal injection of 50 μg Camrelizumab every four days. Representative results from three independent experiments are shown (n = 5). ns, not significant, *p < 0.05, **p < 0.01. PD-1, programmed cell death protein 1; NSG, NOD *Prkdc*^*scid*^ *Il2rg*^*-/-*^; PBMCs, peripheral blood mononuclear cells.

The potential synergistic effects of H101 and anti-PD-1 on tumor suppression were subsequently investigated. U87-MG, T24 and YTS-1 tumor models were established, and humanized immunity was established in these tumors using NSG mice. As the infiltration of immune cells into solid tumors is complex, and to prolong the induction of anti-tumor immune responses, human PBMCs were mixed with tumor cells at a ratio of 1:1, and these were simultaneously subcutaneously injected into NSG mice. Our results demonstrated that H101 treatment significantly inhibited the growth of CAR-positive cells, U87-MG and T24 cells, while anti-PD-1 treatment inhibited the tumor growth of CAR-positive cells, U87-MG and T24 cells. In addition, anti-PD-1 treatment inhibited the growth of all tumor models (Fig. 2A). Notably, the combined therapy of H101 with anti-PD-1 exerted more potent anti-tumor effects than the single agent alone in all the tumor models tested (Fig. 2A). In addition, the combined therapy prolonged the survival of all tumor-bearing mice (Fig. 2B). Results of the immunofluorescence analysis revealed that the combined therapy enhanced the total and IFN-γ-expressing CD8^+^ T cells in tumor tissues (Fig. 2C). These results revealed that H101 synergizes with anti-PD1 may enhance anti-tumor immunity.

### H101 promotes the phagocytosis of macrophages through inhibiting CD47 expression

**Figure 3:**
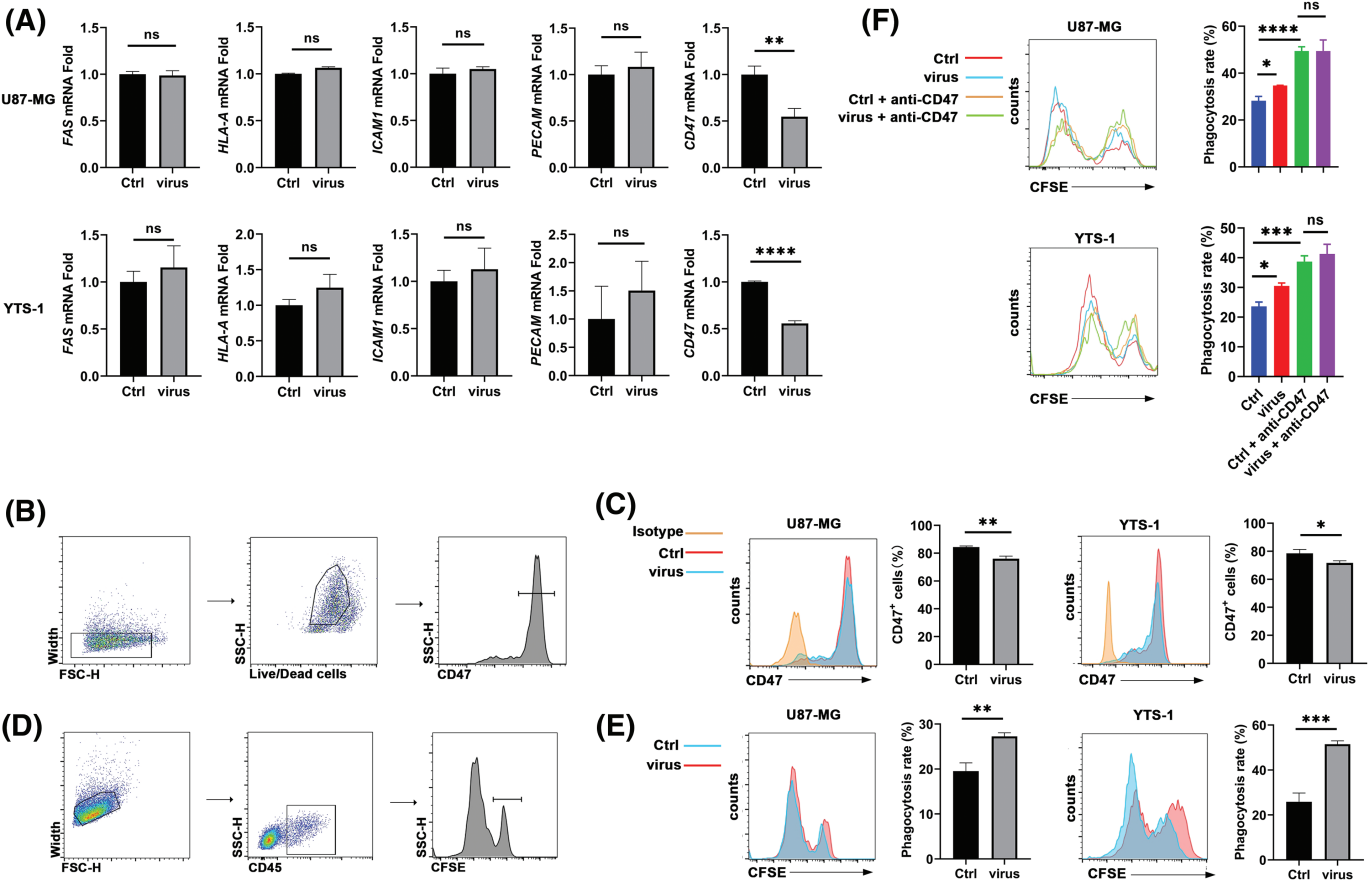
H101 inhibits CD47 expression to induce macrophage phagocytosis. (A) Reverse transcription-quantitative PCR analysis of *FAS*, *ICAM1*, *HLA-A*, *CD47* and *PECAM* mRNA expression in U87-MG and YTS-1 cells infected with H101 at a MOI of 1 for 24 h. (B) Gating strategies. (C) Flow cytometry was used to analyze CD47 on U87-MG and YTS-1 cells infected with oncolytic viruses at a MOI of 1 for 24 h. (D) Gating strategies. (E) Flow cytometry was used to analyze U87-MG or YTS-1 cells infected with H101 at a MOI of 1 for 24 h, phagocytosed by induced THP-1 cells co-cultured for 8 h. (F) Flow cytometry was used to determine the phagocytosis of tumor cells treated with oncolytic viruses at a MOI of 1 for 24 h, co-cultured with induced THP-1 cells following the addition of anti-CD47 (10 μg/mL). Representative results from three independent experiments are shown (n = 3). ns, not significant, *p < 0.05, **p < 0.01, ***p < 0.001, ****p < 0.0001. MOI, multiplicity of infection.

The present study aimed to investigate the impact of H101 on the anti-tumor effects of anti-PD-1. As both U87-MG and T24 cells express CAR, and U87-MG cells exhibit a higher level of CAR expression, U87-MG cells were selected to represent CAR-positive tumors in subsequent experiments. In addition, YTS-1 cells were used to represent CAR-negative tumors. The expression levels of immune-related markers in U87-MG and YTS-1 cells were determined following treatment with H101, and our results demonstrated that the expression levels of FAS, HLA-A and ICAM1 were not changed (Fig. 3A). Subsequently, mRNA expression levels of PECAM and CD47 were investigated, as these proteins play a role in the signals of phagocytes. Following H101 treatment in U87-MG and YTS-1 cells, we found that CD47 expression was significantly reduced following H101 treatment (Fig. 3A). In addition, CD47 protein expression levels were decreased in both U87-MG and YTS-1 cells (Fig. 3B,C). CD47-mediated inhibition of macrophage phagocytosis leads to the loss of the first line of defense against cancer cells [17]. Therefore, we hypothesized that H101 may improve the anti-tumor effects of anti-PD-1, through promoting macrophage phagocytosis of tumor cells. THP-1 cells were induced to differentiate into M0 macrophages, and our results demonstrated that treatment with H101 promoted the induced THP-1 cells to phagocytize U87-MG and YTS-1 cells (Fig. 3D,E). Moreover, CD47 was neutralized with anti-CD47, and our results demonstrated that oncolytic viruses no longer increased the rate of phagocytosis (Fig. 3F). These data suggested that H101 inhibited CD47 expression on the surface of tumor cells, thereby promoting macrophages to phagocytize an increased number of tumor cells.

### Macrophage-activated T cells are activated by H101-treated tumor cells

**Figure 4:**
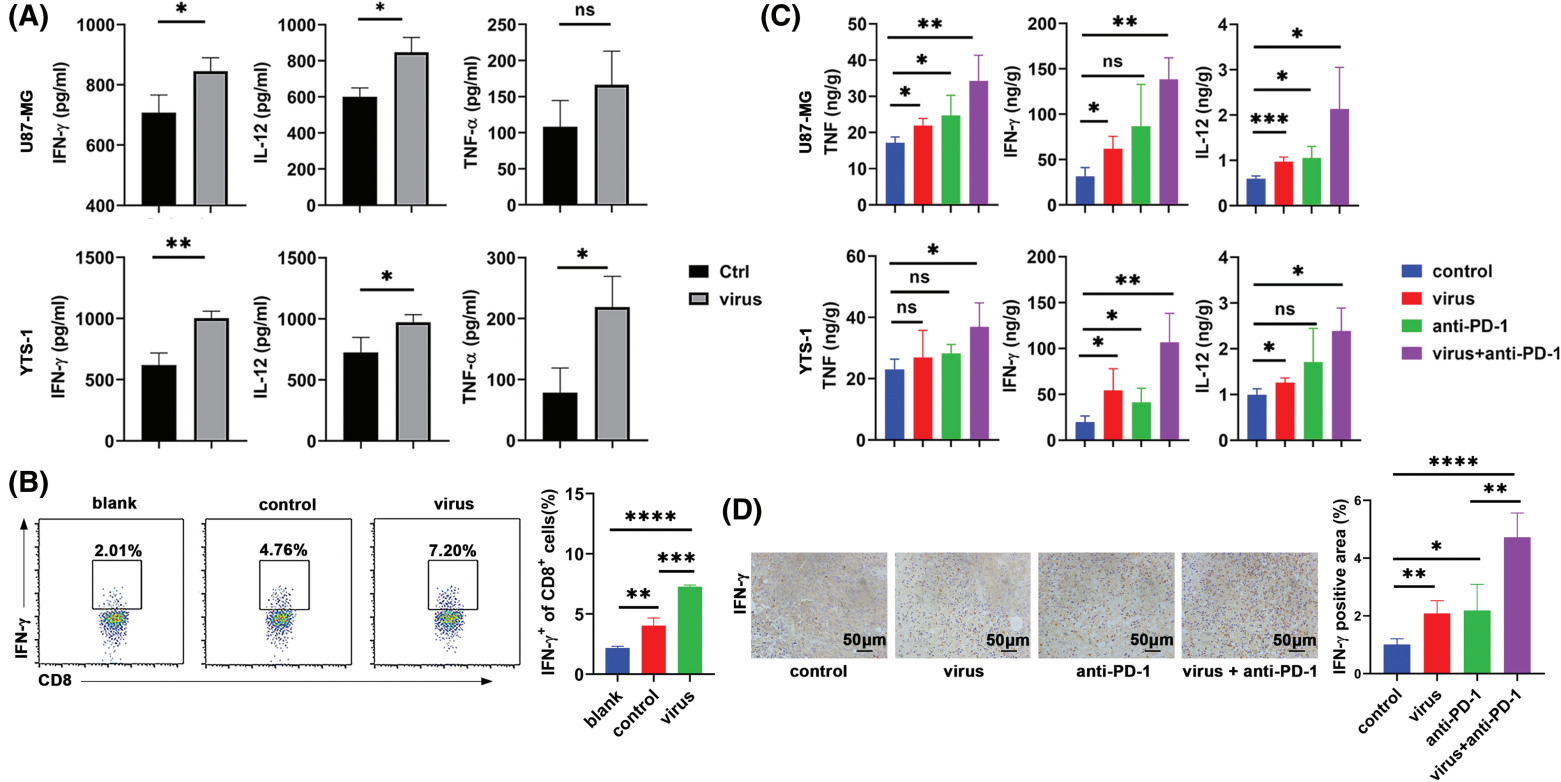
H101-treated tumor cells increase cytokine levels secreted by macrophages to activate T cells. (A) ELISA analysis of TNF, IL-12 and IFN-γ secreted by THP-1 cells co-cultured for 24 h with U87-MG or YTS-1 cells treated with H101 at a MOI of 1.5 for 24 h. (B) Flow cytometry was used to determine the rate of IFN-γ^+^ among CD8^+^ T cells stimulated with supernatants of induced THP-1 cells co-cultured with YTS-1 cells treated with H101 at a MOI of 1.5 for 24 h. (C) ELISA analysis of TNF, IFN-γ and IL-12 levels in tumor tissues of U87-MG and YTS-1 tumor-bearing mice that received the combined treatment of H101 (2.5 × 10^7^ pfu per tumor) with 50 μg of Camrelizumab. (D) Immunohistochemical analysis of IFN-γ in the tumor tissues of YTS-1 tumor-bearing mice that received H101 treatment (2.5 × 10^7^ pfu per tumor) and anti-PD-1 therapy. Representative results from three independent experiments are shown (n = 3). ns, not significant, *p < 0.05, **p < 0.01, ***p < 0.001, ****p < 0.0001. MOI, multiplicity of infection.

Subsequently, the present study aimed to investigate whether increased phagocytosis of tumor cells promotes macrophage activation, thereby enhancing anti-PD-1 effects. Our results demonstrated that induced THP-1 cells, that were co-cultured with H101-treated U87-MG and YTS-1 cells, secreted increased levels of cytokines, including TNF, IL-12 and IFN-γ (Fig. 4A). Following treatment with cell supernatants, CD8^+^ T cells released elevated levels of IFN-γ (Fig. 4B). Subsequently, the levels of TNF, IL-12 and IFN-γ were detected in tumor tissues. Our results demonstrated that the levels of these cytokines were also significantly increased in the lysates of tumor tissues from U87-MG and YTS-1 tumor-bearing mice, following the combined therapy (Fig. 4C). Moreover, results of the IHC analysis demonstrated increased IFN-γ levels in the YTS-1 tumor tissues from mice, following the combined therapy (Fig. 4D). These results suggested that H101 indirectly activated macrophages, and subsequently induced the activation of T cells.

### Macrophage-derived IL-12 plays a role in IFN-γ secretion from T cells

**Figure 5:**
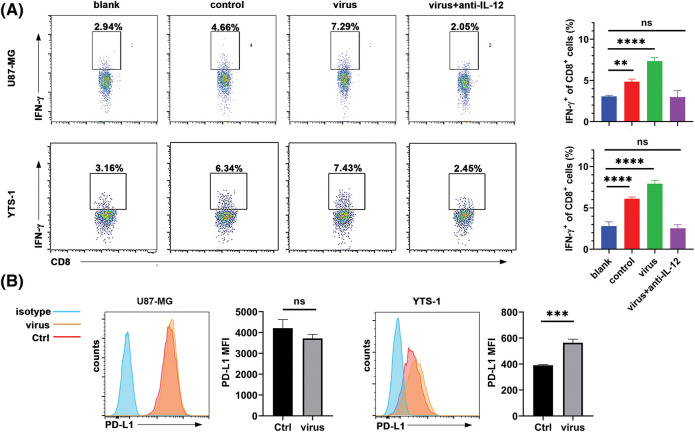
Macrophages promote IFN-γ secretion via IL-12. (A) Flow cytometry was used to determine the frequency of CD8^+^IFN-γ^+^ T cells in T cells stimulated by blank T cell medium (blank), supernatants of induced THP-1 cells (control), supernatants of induced THP-1 phagocytizing oncolytic virus-treated U87-MG or YTS-1 cells without (virus) or with (virus + anti-IL-12) 10 μg/mL anti-IL-12 antibodies. (B) Flow cytometry was used to determine the levels of PD-L1 expressed on U87-MG and YTS-1 cells following H101 treatment at a MOI of 1.5 for 24 h. Representative results from three independent experiments are shown (n = 3). ns, not significant, **p < 0.01, ***p < 0.001, ****p < 0.0001.

IL-12 plays an essential role in the induction of IFN-γ in T cells [18]. Thus, the present study aimed to investigate whether macrophages activated by H101-treated tumor cells promoted IFN-γ secretion from T cells via IL-12. Our results demonstrated that treatment with the anti-IL-12 antibody abolished IFN-γ production from T cells induced by macrophages phagocytizing H101-treated U87-MG and YTS-1 cells (Fig. 5A). Although IFN-γ plays a role in the upregulation of PD-L1 in tumor cells [19], Our results demonstrated that H101 directly upregulated PD-L1 in infected YTS-1 cells (Fig. 5B). Thus, H101 may induce PD-1 signaling activation in T cells, which highlights the requirement for anti-PD-1 treatment following H101 treatment.

## Discussion

The oncolytic virus-induced anti-tumor immune response exhibits potential in cancer therapy [20]. Results of a previous study demonstrated that oncolytic virus exhibits potential in overcoming resistance to immune checkpoint blockade [21]. Moreover, a combination of oncolytic virus and immune checkpoint blockade exhibits potential in the treatment of solid tumors. However, the mechanism by which oncolytic virus induces anti-tumor immunity is yet to be fully elucidated [22]. The present study aimed to investigate the anti-tumor effects of an oncolytic adenovirus, H101, in combination with a PD-1 inhibitor, Camrelizumab, in a humanized immune system mouse model of cancer. Our results demonstrated that the combined therapy of H101 with Camrelizumab resulted in a more significant suppression of tumor growth, compared with either agent alone. Virus-induced anti-tumor immunity arises from the highly destructive nature of virus infection within the tumor, through releasing cellular danger signals and tumor antigens [23]. Thus, the virus-induced cytopathic effects of cancer cell lines were investigated using apoptosis analysis. Our results demonstrated that H101 induced apoptosis in CAR-positive cell lines, U87-MG and T24, but not in CAR-negative cell line, YTS-1. Notably, CAR plays a crucial role in adenoviral infection. In some extent, cancer cells with low levels of CAR expression resist adenoviral infection and oncolysis with dose dependent manner, increased exposure of adenovirus to cancer cells would enhance adenoviral infection and oncolysis [16]. Thus, H101 inhibited tumor growth and prolonged survival in the CAR-positive subcutaneous tumor model, compared with the CAR-negative tumor model. However, regardless of CAR expression, combining H101 with systemic PD-1 blockade resulted in the inhibition of synergistic tumor growth and prolonged the survival of tumor-bearing mice, compared with treatment with each agent alone. Immunofluorescence analysis revealed increased IFN-γ-expressing CD8^+^ T cells in the combined treatment group, compared with tumors treated with either H101 or PD-1 blockade alone, confirming the enhanced anti-tumor response exerted by IFN-γ-expressing CD8^+^ T cells. These results suggested that H101 infection recruits CD8^+^ T cells into the tumor microenvironment, and PD-1 blockade further activates CD8^+^ T cells to attack the tumor. Results of a previous study revealed that tumor-infiltrating T cells are associated with response to anti-PD-1 treatment. Patients without response to PD-1 blockade therapy were more likely to lack CD8^+^ T cells inside the tumor lesions [24]. Thus, increasing the levels of CD8^+^ T cells in tumors using oncolytic viruses may improve the anti-tumor activity of PD-1 blockade therapy. Thus, H101 combined with PD-1 blockade may exhibit potential as a strategy for immunotherapy. Results of previous studies revealed that oncolytic virus-induced upregulation of PD-L1 in the tumor microenvironment synergized with anti-PD-L1 treatment, leading to improved therapeutic efficacy [25,26]. In addition, results of a further study demonstrated that localized oncolytic infection abrogated resistance to systemic anti-PD-1 immunotherapy, through upregulating PD-L1 in tumor cells and eliciting a broad-range T-cell attack against the neoantigen [27].

The mechanisms underlying virus-triggered anti-tumor immune responses are yet to be fully elucidated. In the present study, immune-related markers were detected in H101-treated cells, and the results demonstrated that viral infection resulted in the downregulation of CD47 in infected cancer cells, which promoted THP-1 cell-induced phagocytosis of cancer cells. Our study demonstrated that H101 may promote macrophages to phagocytose viral-infected tumor cells through inhibiting CD47 on the tumor cell surface. Notably, CD47 plays an important role in tumor escape from macrophage-mediated phagocytosis [28]. Thus, blocking CD47 expression on cancer cells may enhance the anti-tumor immune response. Strategies that target CD47, including the use of the anti-CD47 antibody [29] and CD47 knockdown [30] for cancer therapy, demonstrated that CD47 blockade significantly increased macrophage infiltration and phagocytosis, thereby enhancing the anti-tumor response. As CD47 also exists in healthy cells, anti-CD47 antibodies may damage these cells and cause potential off-target effects, such as anemia, thrombocytopenia and leukopenia; thus, limiting clinical use [31,32]. In addition, H101-induced inhibition of CD47 occurs only in tumors due to selective replication of H101 in tumor cells. Other reports demonstrated that macrophages may induce CD8^+^ T cells responses in mice upon CD47 blockade [33]. Thus, we hypothesized that H101-induced downregulation of CD47 may activate T-cell immunity through macrophages. The effects of macrophage-mediated T cell responses were evaluated in H101-treated cells. Our results demonstrated that co-culturing of induced THP-1 cells with H101-treated cancer cells produced an increased expression of cytokines, including TNF, IL-12 and IFN-γ. Moreover, following the addition of supernatants from macrophages to CD8^+^ T cells, the expression levels of of IFN-γ were observed. In addition, increased expression levels of TNF, IL-12 and IFN-γ were observed in tumor tissues treated with H101 or PD-1 blockade. Combining H101 with PD-1 blockade resulted in a further increase of these cytokines, compared with tumors treated with a single agent. These results confirmed the macrophage-mediated activation of CD8^+^ T cells by both H101 or PD-1 blockade. As macrophages express PD-1, activation of PD-1 signaling on tumor-associated macrophages using PD-L1 may suppress the ability of PD-1^+^ macrophages to phagocytose tumor cells [34]. PD-1 blockade may induce macrophages to phagocytose tumor cells and activate CD8^+^ T cells. Strategies that combine immune checkpoint PD-1/PD-L1 blockade with innate immune checkpoint CD47/SIRPα blockade exhibit potential in cancer immunotherapy [35–37]. Our study demonstrated that a combination therapy of H101 with PD-1 blockade significantly inhibited tumor growth and prolonged survival in a humanized immune system mouse tumor model. IL-12 plays an essential role in the induction of IFN-γ in T cells [18]. Our study demonstrated that removing macrophage-derived IL-12 using anti-IL-12 antibodies abolished IFN-γ from T cells. These results revealed that macrophage-derived IL-12 induced IFN-γ secretion in T cells. Moreover, IFN-γ may induce the upregulation of PD-L1 in tumors [19]. Notably, our study demonstrated that H101 infection induced the upregulation of PD-L1 in YTS-1 cells, and induced PD-1 signaling activation in T cells, supporting the use of the combined therapy with PD-1 blockade. Results of previous studies also demonstrated that infection with oncolytic viruses led to the upregulation of PD-1 in tumor cells and tumor-infiltrating immune cells, and the combination therapy of intratumorally administered oncolytic viruses and systemic PD-1 or PD-L1 blockade resulted in a marked enhancement of the anti-tumor immune effect, leading to rejection of the oncolytic virus-treated and distant, non-infected tumors [25,38]. Our study encourage the use of combined therapy with H101 and PD-1 or PD-L1 blockade in treating cancer. In fact, we conducted a clinical trial to evaluate efficacy and safety of intravesical instillation of H101 combined with camrelizumab in the treatment of bladder cancer (NCT05564897).

There are several limitations in our study. First, we use the humanized mouse models to evaluate the efficacy of immunotherapies. Althourgh humanized mouse models are commonly used for human immuno-oncology research, it has some shortcomings, mainly including the limited development of mature innate immune cell populations (monocytes, macrophages, conventional and plasmacytoid dendritic cells (DCs), and natural killer (NK) cells). Second, we did not establish an orthotopic tumor model to investigate the anti-tumor effects of H101 combined with camrelizumab. Orthotopic tumor model can better simulate the microenvironment of tumor growth *in vivo*, providing an accurate model for predicting drug efficacy.

## Conclusions

In conclusion, Our study demonstrated that H101 acts synergistically to enhance the therapeutic efficacy of PD-1 blockade in cancer through suppressing CD47 signaling. Notably, this may induce macrophages to phagocytose tumor cells and activate CD8^+^ T cells. These findings support the key clinical implications of the combination of H101 and PD-1 blockade in cancer immunotherapy.

## Data Availability

The datasets used and/or analyzed during the current study are available from the corresponding author on reasonable request.
